# Supplementary material for: ARPC1B Is Associated with Lethal Prostate Cancer and Its Inhibition Decreases Cell Invasion and Migration In Vitro
Source: Int J Mol Sci. 2022 Jan 27;23(3):1476. doi: 10.3390/ijms23031476 (PMC8836051; doi:10.3390/ijms23031476)
Supplement: Supplementary file 1 [file ijms-23-01476-s001.zip › ijms-1561704 (supp) (2)/Supp_Table S1.pdf]

No	Antibody	Catalog number	Supplier	WB dilution
1	Rabbit anti-ARPC1B antibody	HPA004832-100UL	Sigma-Aldrich	1:1000
2	Mouse anti-GAPDH antibody	60004-1-Ig	ProteinTech	1:1000
3	Mouse anti-beta actin antibody	4967S	Cell Signaling	1:1000
4	Rabbit Monoclonal anti-ERG	97249S	Cell Signaling	1:1000
5	Mouse Monoclonal anti-N-Cadherin	Sc-393933	Santa Cruz Bio	1:1000
6	Rabbit Monoclonal anti-E-Cadherin	ab40772	abcam	1:1000
7	Rabbit polyclonal anti-Lamin A	Sc- 20680	Santa Cruz Bio	1:500
8	Cyclin B1	ab32053	abcam	1:1000

9	Aurora A kinase	11-8099	Abeomics	1:500
10	Goat anti-Rabbit IgG antibody	7074S	Cell Signaling	1:1000
11	Goat anti-Mouse IgG antibody	7076S	Cell Signaling	1:1000

Supplementary table S1 List of Antibodies
