# Supplementary figures and images for: ARPC1B Is Associated with Lethal Prostate Cancer and Its Inhibition Decreases Cell Invasion and Migration In Vitro
Source: Int J Mol Sci. 2022 Jan 27;23(3):1476. doi: 10.3390/ijms23031476 (PMC8836051; doi:10.3390/ijms23031476)

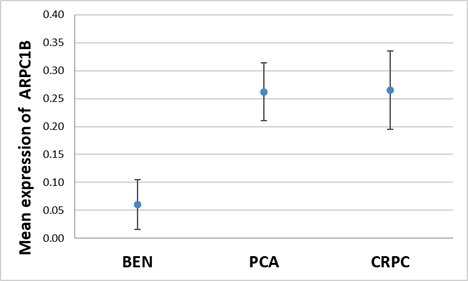

Supplement: Supplementary file 1 [file ijms-23-01476-s001.zip › ijms-1561704 (supp) (2)/Supplementary Figure S1.tif]

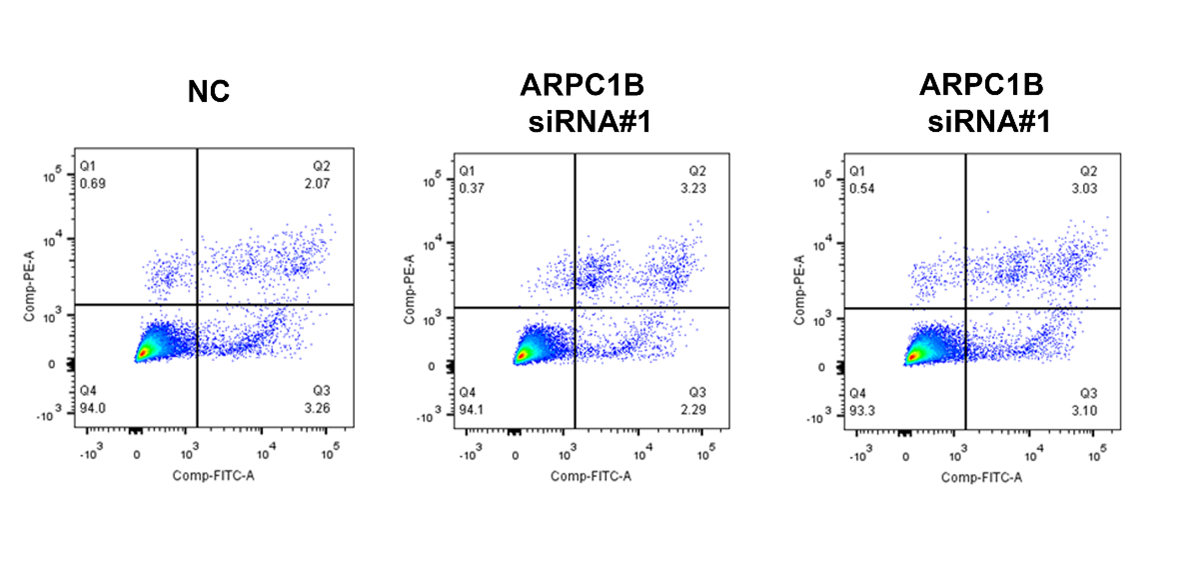

Supplement: Supplementary file 1 [file ijms-23-01476-s001.zip › ijms-1561704 (supp) (2)/Supplementary Figure S2.tif]
